# Effects of Acute Physical Fatigue on Gaze Behavior and Performance During a Badminton Game

**DOI:** 10.3389/fspor.2021.725625

**Published:** 2021-10-05

**Authors:** Mildred Loiseau-Taupin, Alexis Ruffault, Jean Slawinski, Lucile Delabarre, Dimitri Bayle

**Affiliations:** ^1^Laboratory Sport, Expertise, Performance (EA7370), French Institute of Sport (INSEP), Paris, France; ^2^Unité de Recherche Intrafacultaire Santé et Société, Université de Liège, Liège, Belgium; ^3^LICAE Lab, UFR STAPS, University of Paris, Nanterre, France

**Keywords:** physiological load, eye movements, visual search strategy, visual perception, racket sports

## Abstract

In badminton, the ability to quickly gather relevant visual information is one of the most important determinants of performance. However, gaze behavior has never been investigated in a real-game setting (with fatigue), nor related to performance. The aim of this study was to evaluate the effect of fatigue on gaze behavior during a badminton game setting, and to determine the relationship between fatigue, performance and gaze behavior. Nineteen novice badminton players equipped with eye-tracking glasses played two badminton sets: one before and one after a fatiguing task. The duration and number of fixations for each exchange were evaluated for nine areas of interest. Performance in terms of points won or lost and successful strokes was not impacted by fatigue, however fatigue induced more fixations per exchange on two areas of interest (shuttlecock and empty area after the opponent's stroke). Furthermore, two distinct gaze behaviors were found for successful and unsuccessful performance: points won were associated with fixations on the boundary lines and few fixation durations on empty area before the participant's stroke; successful strokes were related to long fixation durations, few fixation durations on empty area and a large number of fixations on the shuttlecock, racket, opponent's upper body and anticipation area. This is the first study to use a mobile eye-tracking system to capture gaze behavior during a real badminton game setting: fatigue induced changes in gaze behavior, and successful and unsuccessful performance were associated with two distinct gaze behaviors.

## Introduction

In racket sports, the ability to rapidly gather relevant visual information is one of the main determinants of sports performance. This active process is essential for effective motor action responses (Abernethy and Russell, 1987; Abernethy et al., 1993). A good visual acuity, a wide field of vision, and a good recognition of peripheral elements allow players to anticipate events (Afonso et al., 2012; Alder et al., 2019). This is particularly true in badminton, which is the fastest racket sport in terms of flight velocities (Alder et al., 2019) and is characterized by short, high intensity actions (Alder et al., 2014). The study of gaze behavior during sport is challenging, however mobile eye-tracking systems can be used to gather relevant data (Abernethy et al., 1993).

Studies have shown that the number and duration of fixations (Aziz, 2017) and their locations (Barreto et al., 2021) are task and context specific. Despite this, 69% of eye tracking studies have been conducted in laboratory conditions (Abernethy et al., 1993). Although such studies provide useful information, the controlled environments, constrained cues, few response possibilities, or the evaluation of discrete parts of an action limit the conclusions which can be drawn (Borg, 1982; Belza, 1994; Blomqvist et al., 2001; Brams et al., 2019). To fully understand gaze behavior during matches, studies must be conducted in real-game situations. The purpose of gaze behavior is to take in information from different areas by the use of a search strategy (Abernethy et al., 1993). Behavior can be characterized by variables such as the duration and number of fixations. Studies have demonstrated that gaze behaviors differ according to the study conditions, for example in representative task designs vs. well-controlled laboratory experiments (Barreto et al., 2021), and that behavior must be evaluated in real settings involving a variety of situations (Brenner and Smeets, 2017) which are ecologically valid (Casanova et al., 2013). Furthermore, gaze behavior can only be fully understood if it is related to performance (Chia et al., 2017). However, to date, this relationship has been little studied in real badminton conditions, although it has been evaluated during specific actions in basketball (Cohen, 1988), karate (Connor et al., 2018), sailing (Dicks et al., 2010) and gymnastics (Faber et al., 2012). To our knowledge, only one study in racket sports linked gaze behavior to performance using a mobile eye-tracking system, however this was in a strictly controlled tennis situation (i.e., four types of serves) that was very different to the a real-game setting. Four areas of interest were mapped: ball before bounce, bounce area, ball after bounce and other. The results showed that successful strokes were related to longer fixation durations during the first part of the ball flight (Gegenfurtner et al., 2011).

One feature of the real-game situation that is not present in controlled studies is fatigue. Fatigue has been defined as a decrease in physical performance that is associated with an increase in the real or perceived difficulty of a task (Hagemann et al., 2006). Fatigue impacts on the interactions between cognitive processes and motor actions (Hughes et al., 1993; Hüttermann et al., 2018) and can impair technical skills such as gaze behavior, movement speed, or tactical choices (Kassner et al., 2014; Kredel et al., 2017; Kolman et al., 2019). Furthermore, it impacts perceptual-cognitive processes differently depending on the physical intensity, the type of physical exercise, and the motor task (MacIntosh, 2006). However, the impact of fatigue on gaze behavior in racket sports has only been evaluated in one, reasonably representative, study. In a badminton task that involved responding to blocks of actions displayed on a screen (shadow shots and verbal confirmations) interspersed with a physical fatiguing task, participants were able to sustain the additional mental effort induced by physical fatigue during the main part of the exercise, but not at the end: during the last block, the number of fixations increased, their durations shortened (i.e., less effective gaze behavior), and the accuracy of the motor responses decreased (Mann et al., 2007).

These studies provided some information about gaze behavior and the impact of fatigue on gaze behavior, however, to the best of our knowledge, no studies have yet evaluated the impact of fatigue on gaze behavior or the relationship between fatigue, gaze behavior and performance in a real-game setting.

This study aimed to explain visual perception in non-experienced racket sport players. The first aim was therefore to determine the relationship between fatigue and gaze behavior during a badminton game and the second aim was to determine the relationship between gaze behavior, fatigue and performance. We hypothesized that acute physical fatigue would reduce fixation duration and increase the number of fixations per rally (Alder et al., 2019), and that fixation duration would be shorter and the number of fixations on the shuttlecock (providing relevant information for novices) would be higher for successful compared to unsuccessful strokes.

## Materials and Methods

### Participants

Nineteen right-handed individuals (mean age = 26.0 ± 2.9 years, eight women, mean age = 27.3 ± 2.0) voluntarily participated in this study. None had any experience in any racket sports. We chose novice participants in order to seek a relationship between the gaze behavior variables analyzed and performance in non-experienced players. A power analysis for mixed models was performed using a procedure developed by Westfall et al. (2014). The design parameters were the effect size (set at 0.5), the α value (set at 0.05), the statistical power (set at 0.8) and the number of participants (set at 19). The power analysis revealed that a minimum of 48.7 stimuli were required. In our study, the number of stimuli was 73.7 ± 11.6 (i.e., number of exchanges per participant).

Participants were first informed of the study procedure and were then screened for eligibility. All participants provided written informed consent for participation and the study was approved by a National Ethical Committee (CER-Paris-Saclay-2019-058).

### Apparatus and Measures

#### Gaze Behavior

A binocular mobile eye-tracking system (Pupil Labs Core eye-tracker, accuracy 0.6°, precision 0.02°) was used to record eye motion. It was a wearable mobile eye tracking headset with one scene camera (120 Hz) and two infrared spectrum eye cameras for dark pupil detection (200 Hz). The mobile eye-tracking system was calibrated for each participant at the start of the session and recalibrated between each game point, using screen marker calibration methods with five markers (Milazzo et al., 2016). For the recalibration, participants were asked to look at a single marker fixed on a post positioned 1.98 m directly in front of their eyes.

#### Performance Indicators

The sessions were video recorded with a JVC GC PX100 BEU Camcorder (frequency: 100 Hz) placed in a fixed position behind and to the right of the participant (on the same side of the court as the participant). This position allowed the whole field to be captured (height: 2.20 m, distance from the back boundary line: 4.05 m).

#### Perceived Fatigue and Exertion

Perceived fatigue was assessed using a visual analog scale ranging from 0 (no fatigue) to 10 (maximal fatigue and exhaustion), which captures momentary fatigue and is validated for physical activity (Murray and Hunfalvay, 2017). Perceived exertion was assessed with the Borg scale which ranges from 6 (no exertion) to 20 (maximal exertion) (Nakashima and Kumada, 2017) and captures participants' perception of exercise intensity.

#### Heart Rate

Each participant wore a Polar RS400 running computer to measure heart rate and to ensure that they reached around 90% of their maximal theoretical heart rate during the fatigue session, corresponding to the average heart rate during a badminton match (Alder et al., 2019).

### Experimental Procedure

All participants played two badminton sets against the same opponent who was a racket (not badminton) player. Only one experiment was performed per day to avoid fatiguing the opponent. The experimental procedure included three phases: (i) a pre-fatigue badminton set; (ii) a fatigue session, and (iii) a post-fatigue badminton set. Perceived fatigue and exertion were measured after each phase.

#### Badminton Game

Each badminton set was played to 21 points in accordance with the Badminton World Federation rules. Participants were equipped with a binocular mobile eye-tracking system and the games were recorded with a camcorder.

#### Fatigue Session

A specific fatigue session was designed by the national badminton coach. The session lasted 18 min and included two series of 12 different exercises (20 s of running and 20 s of rest), with 2 min of rest between each. Each series was performed in a half-court and incorporated shadow training with a racket. Participants were instructed to sprint as fast as possible in order to reproduce the same level of fatigue as in a match. They wore a Polar RS400 running computer for the whole procedure to record heart rate. The mean percentage of heart rate was 93.0 ± 4.1 % during this fatigue session.

### Data Processing

Mean heart rate was calculated as a percentage of the maximal theoretical heart rate for each experimental phase. Maximal theoretical heart rate was calculated using the formula by Milazzo et al. (2016). Mean perceived fatigue and exertion scores were calculated for each session.

**Figure 1:**
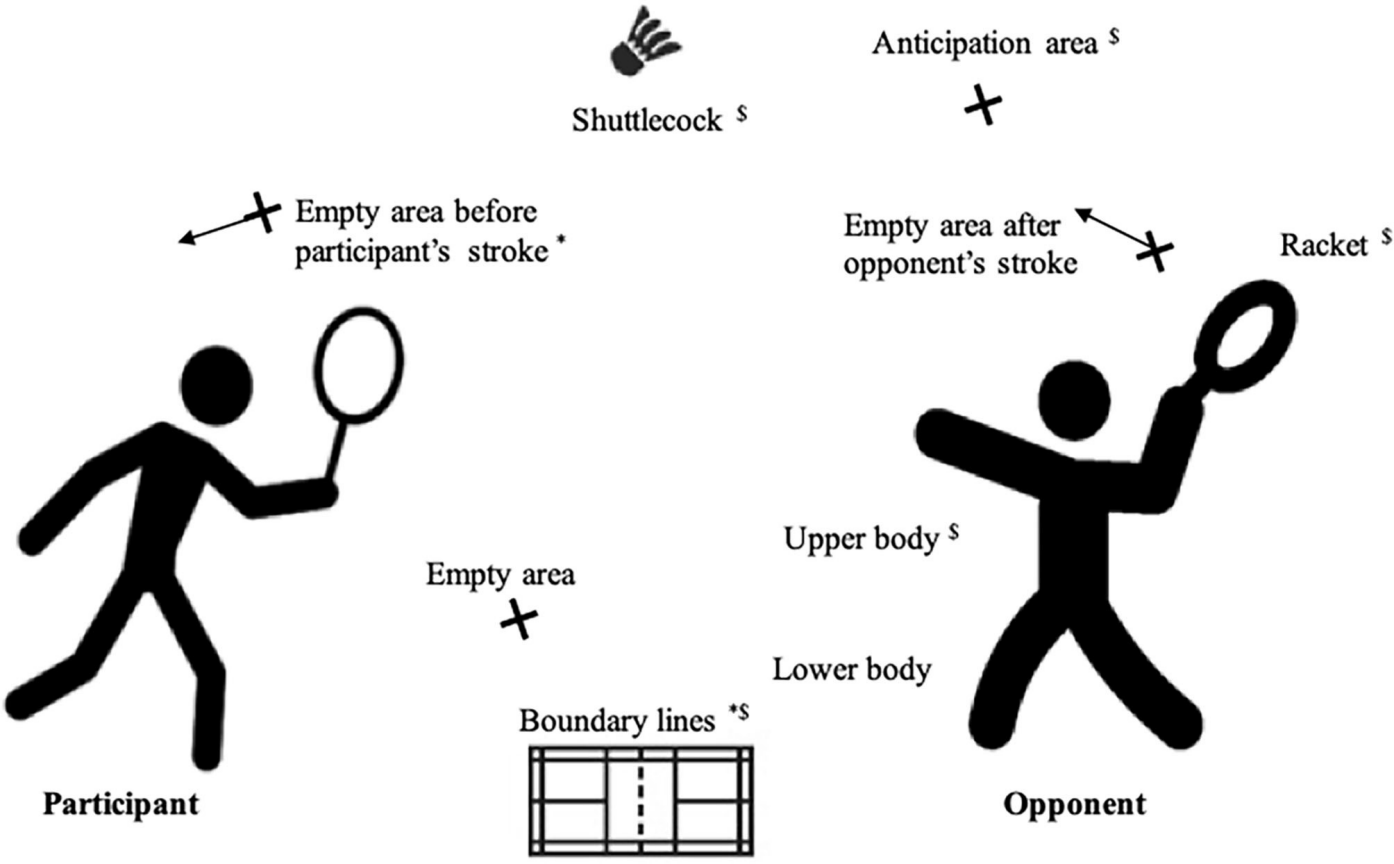
Nine areas of interest analyzed for gaze behaviors. *Areas of interest involved in performance for win point. ^$^Areas of interest involved in performance for successful stroke.

We determined nine areas of interest (AOIs), based on the study by Phomsoupha and Laffaye (2015): shuttlecock, opponent's racket, opponent's upper body, opponent's lower body, boundary lines (court limits), anticipation area (anticipation of the contact point between shuttlecock and opponent's racket), empty area after opponent's stroke (while the participant located the shuttlecock after the opponent's stroke), empty area before participant's stroke (no location before the participant's stroke), empty area (no discernible location) (Figure 1). We coded 10% of the eye-tracking data by two independent sport scientists. An analysis of the degree of agreement was conducted and we obtained 84.5% of concordance for AOIs. The error of accuracy (0.6°) corresponds to a maximum imprecision of 14 cm at a distance of 13.40 m (distance between the two background lines; tan(0.6) x 1340 = 14), and 4.14 cm at a distance of 3.96 m (distance between the two serve lines; Tan(0.6) x 396 = 4.14).

Opponent's and participant's serves and strokes during rallies were considered separately (mean number of exchanges per participant was 73.7 ± 11.6). Gaze behavior was only evaluated during strokes. Rallies were composed of several exchanges; an exchange was defined as a stroke by the opponent followed by a stroke by the participant.

**Figure 2:**
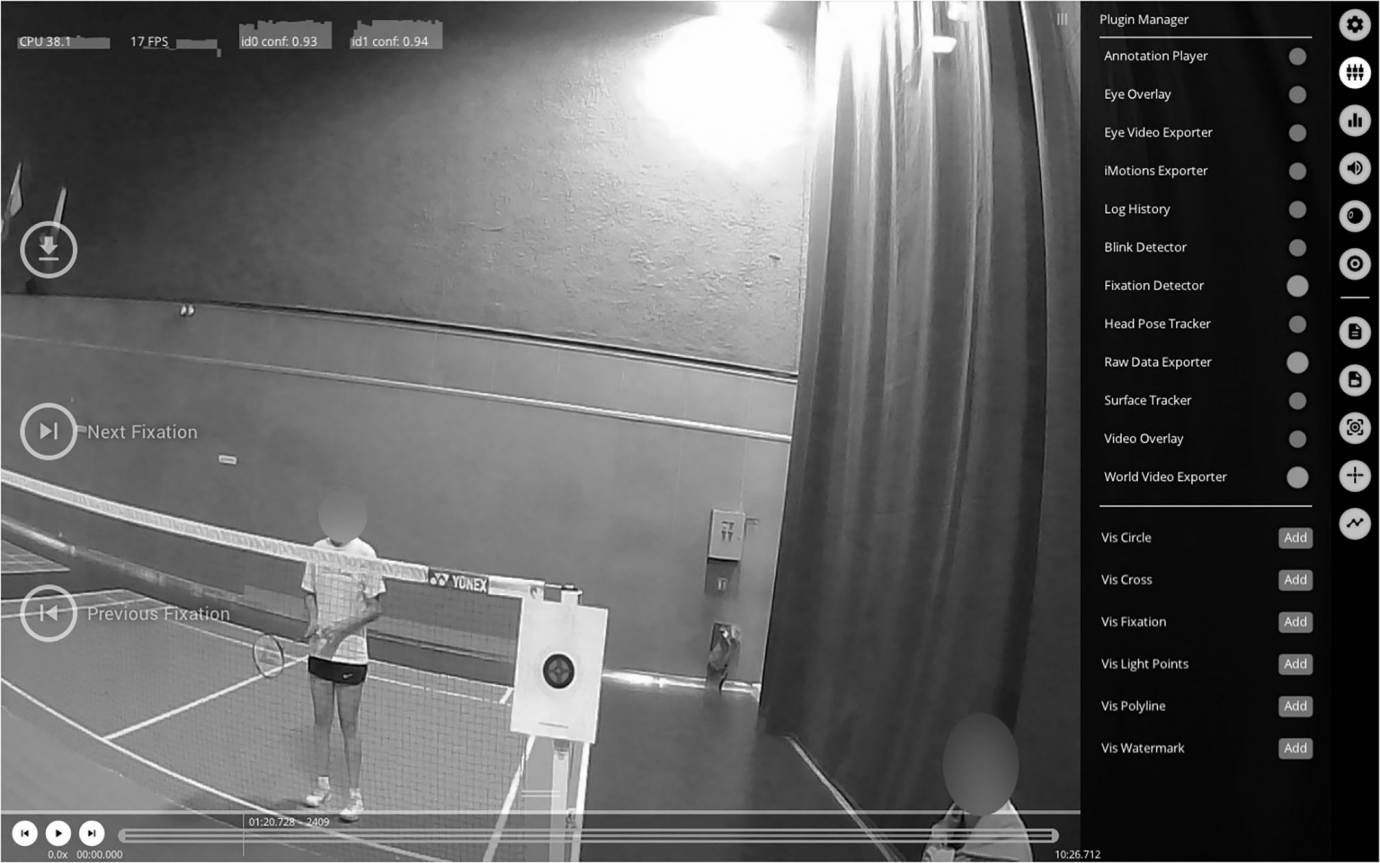
Gaze point during single marker calibration obtained on Pupil Player software.

Pupil Player software was used to identify gaze location from the mobile eye-tracker recordings of eye and head motion. Pupil Capture software recorded and mapped pupil positions from eye to scene using a pupil detection algorithm and then a transfer function (Mann et al., 2007). Thus, one gaze point was obtained for each time instant: we termed this fixation for simplicity (Figure 2). We manually determined, frame by frame, which of the nine AOIs defined above was fixed for each time instant of the world camera video recording by the eye-tracker using Pupil Player software. The timestamp of the first video frame in one of the nine AOI is defined as the beginning of the fixation. Timestamp of the last frame of consecutive fixation in the same AOI is defined as the end of the fixation

Five variables were recorded for each participant during the badminton sets: mean fixation duration, number of fixations and number of switches per exchange; and mean fixation duration and total number of fixations for each AOI per set. A switch was defined as a shift in the gaze between two AOIs. The total number of fixations was the sum of fixations per AOI per set divided by the number of rallies in the set.

The number of points, strokes performed, and faults were determined from the video recording of the badminton set and Kinovea 0.8.15 Software. Success was defined using 2 criteria: points won/lost and successful strokes. Points were calculated for each rally. A point was won if the participant scored the point and lost if the participant committed a fault. A stroke was successful when the participant hit the shuttlecock to the opponent's side without a fault, or unsuccessful if the participant did not touch the shuttlecock or if the shuttlecock did not land on the opponent's side of the court.

### Statistical Analysis

Data were analyzed using RStudio Version 1.4.1106. For each participant and each badminton set, a mean 0.8 ± 1.0% of fixation duration data were removed using univariate anomaly detection. We then removed fixation durations that were more than two standard deviations longer than the mean duration: higher than 1047 ms for the pre-fatigue sets and higher than 1043 ms for the post-fatigue sets (a mean 5.1 ± 5.0% of data were deleted). The number of observations corresponded to the total number of fixations in the pre-fatigue (4,797) and post-fatigue sets (5,015) (t_18_ = −0.78, *p* > 0.05, Cohen's *d* = −0.18).

Linear mixed models were performed to analyze the following variables: fatigue (two conditions: pre-post fatigue sets), performance (two conditions: points won and lost or successful and unsuccessful strokes), gaze behavior (fixation duration, number of fixations, number of switches, fixation duration and number of fixations per AOIs) and fatigue markers (heart rate, perceived fatigue and perceived exertion) with participants as a random effect for all analyses.

We first compared fatigue markers across the experimental phases and then estimated the effects of fatigue on performance and on gaze behavior. Finally, we estimated the effects of performance on gaze behavior.

For all mixed linear models, significance was set at *p* < 0.05 a and conditional R^2^ value was used to indicate the model's total explanatory power. Tukey *post-hoc* tests to determine specific differences for the significant mixed linear models. The strength of the effect was calculated using Cohen's *d* and was interpreted as small for values around 0.2, medium around 0.5 and large for those around 0.8 (Piras et al., 2014).

## Results

### Fatigue Levels

Table 1 displays the three markers of fatigue. There was a main effect of heart rate (*p* < 0.001, *R*^2^ = 0.76), perceived fatigue (*p* < 0.001, *R*^2^ = 0.77) and perceived exertion (*p* < 0.001, *R*^2^ = 0.67) between the phases of the experimental procedure. *Post-hoc* tests on fatigue indicators demonstrated that fatigue increased between the pre-fatigue and post-fatigue sets (all *p* values < 0.01), and between the pre-fatigue sets and fatigue sessions (all *p* values < 0.001), and decreased between the fatigue sessions and post-fatigue sets (all *p* values < 0.001).

**Table 1:** Mean percentage of maximal heart rate during each session, mean perceived fatigue and mean exertion with Borg scale after each session.

	**Set 1—Pre-Fatigue**	**Fatigue Protocol**	**Set 2—Post-Fatigue**
Heart rate (%)	77.6 ± 10.5	93.0 ± 4.1^***^	85.9 ± 7.9^**^^,^ ^$$$^
Perceived Fatigue	4.1 ± 1.1	7.8 ± 0.9^***^	5.9 ± 1.2^**^^,^ ^$$$^
Borg Scale	12.0 ± 1.6	16.6 ± 1.5^***^	13.6 ± 2.3^**^^,^ ^$$$^

*Scores are presented as mean ± standard deviation. Heart rate is in percentage of maximal heart rate. Perceived fatigue and Borg scale are a number from each visual analog scale*.

** *p < 0.01,*

*** *p < 0.001 from Pre-Fatigue Sets;*

$$$ *p < 0.001 from Fatigue Protocol*.

### Effects of Fatigue on Performance

There was no effect of fatigue on either points won/lost (*p* = 0.88, *R*^2^ = not applicable) or successful/unsuccessful strokes (*p* = 0.47, *R*^2^ = 0.78).

### Effects of Fatigue on Gaze Behaviors

There was no effect of fatigue on fixation duration (*p* = 0.16, *R*^2^ = 0.50, Cohen's *d* = 0.34), number of fixations (*p* = 0.14, *R*^2^ = 0.27, Cohen's *d* = −0.43) or the number of switches (*p* = 0.12, *R*^2^ = 0.43, Cohen's *d* = −0.40). There was no effect of fatigue on fixation duration for any of the nine AOIs (all *p* values > 0.05). There was a significant effect of fatigue on the number of fixations for shuttlecock (*p* < 0.05, *R*^2^ = 0.61, Cohen's *d* = −0.53) and empty area after opponent's stroke (*p* < 0.05, *R*^2^ = 0.48, Cohen's *d* = −0.51): the number of fixations on the shuttlecock was lower in pre- (144.4 ± 28.3) than post-fatigue sets (159.1 ± 27.2) and the number of fixations on empty area after the opponent's stroke was lower in pre- (45.6 ± 23.3) than post-fatigue sets (56.0 ± 17.6).

### Gaze Behavior and Performance

#### Performance and Number of Fixations

##### Point Won/Lost

There was no effect of the number of fixations on performance (*p* = 0.79, *R*^2^ = 0.19, Cohen's *d* = −0.05). For the number of fixations per AOI, the only main significant effect on performance was for the boundary lines (*p* < 0.05, *R*^2^ = 0.25, Cohen's *d* = 0.42): the number of fixations on the boundary lines was higher for points won (9.1 ± 10.3) than lost (5.4 ± 7.0).

##### Successful/Unsuccessful Strokes

There was an effect of the number of fixations on performance (*p* < 0.001, *R*^2^ = 0.50, Cohen's *d* = 0.69): the number of fixations was higher for successful (3.2 ± 0.5) than unsuccessful strokes (2.3 ± 0.4). There was a significant effect of the number of fixations on performance for five locations: opponent's racket (*p* < 0.001, *R*^2^ = 0.49, Cohen's *d* = 1.75), shuttlecock (*p* < 0.001, *R*^2^ = 0.56, Cohen's *d* = 1.27), opponent's upper body (*p* < 0.001, *R*^2^ = 0.66, Cohen's *d* = 2.0), anticipation area (*p* < 0.001, *R*^2^ = 0.77, Cohen's *d* = 3.1) and boundary lines (*p* < 0.001, *R*^2^ = 0.30, Cohen's *d* = −1.15). The number of fixations was higher for successful than unsuccessful strokes: opponent's racket: 13.4 ± 9.7 vs. 1.0 ± 3.1, shuttlecock: 181.0 ± 48.8 vs. 126.6 ± 33.8, opponent's upper body: 27.1 ± 13.6 vs. 4.0 ± 5.7 and anticipation area: 67.2 ± 22.9 vs. 8.4 ± 11.2. However, for the boundary lines, the number of fixations was lower for successful than unsuccessful strokes (2.0 ± 2.6 vs. 10.9 ± 10.5) (Table 2).

**Table 2:** Number of fixations on each location for successful and unsuccessful strokes during rallies.

**Dependent variable Locations**	**Successful strokes**	**Unsuccessful strokes**	**P value**	**Fixed effect estimate (95% CI)**	**Effect size (95% CI)**	**R^**2**^**
Shuttlecock	181.0 ± 48.8	126.6 ± 33.8	^***^	−54.4 (−69.4; −39.4)	1.3 (0.8; 1.7)	0.56
Opponent's Racket	13.4 ± 9.7	1.0 ± 3.1	^***^	−58.8 (−66.3, −51.3)	3.1 (2.2; 4.0)	0.77
Opponent's Upper body	27.1 ± 13.6	4.0 ± 5.7	^***^	−23.1 (−27.2; −19.0)	2.0 (1.4; 2.6)	0.66
Opponent's Lower body	1.6 ± 3.2	0.2 ± 1.0	N/A	N/A	N/A	N/A
Boundary lines	2.0 ± 2.6	10.9 ± 10.5	^**^	8.9 (5.6; 12.2)	−1.2 (−1.7; −0.6)	0.30
Anticipation area	67.2± 22.9	8.4 ± 11.2	^***^	−58.8 (−66.3;−51.3)	3.2 (2.2; 4.0)	0.77
Empty area after opponent's stroke	31.9 ± 17.6	32.6 ± 14.1	ns	0.6 (−5.5; 6.7)	−0.04 (−0.3;0.3)	0.29
Empty area before participant's stroke	57.3 ± 24.2	54.3 ± 22.3	ns	−3.0 (−10.9; 4.8)	0.1 (−0.1;0.4)	0.45
Empty area	3.2 ± 12.2	7.2 ± 12.0	ns	4.0 (−0.3; 8.3)	−0.3 (−0.5; −0.2)	0.40

*Scores are presented as mean ± standard deviation*.

*p < 0.05,

** *p < 0.01,*

*** *p < 0.001, ns: Not Significant, N/A: Not Applicable*.

#### Performance and Fixation Durations

##### Points Won/Lost

There was no overall effect of fixation duration on performance (*p* = 0.89, *R*^2^ = 0.17, Cohen's *d* = 0.03). Analysis of each AOI showed that only the fixation duration for the empty area before the participant's stroke impacted performance (*p* < 0.05, *R*^2^ = 0.48): fixation duration was shorter for points won (64.6 ± 45.3 ms) than points lost (79.7 ± 35.6 ms).

##### Successful/Unsuccessful Strokes

There was an effect of fixation duration on performance (*p* < 0.001, *R*^2^ = 0.64, Cohen's *d* = 2.00): fixations were longer for successful (364.2 ± 36.3 ms) than unsuccessful strokes (336.2 ± 43.4 ms). Analysis of each AOI showed an effect of fixation duration on performance for three AOIs: opponent's upper body (*p* < 0.001, *R*^2^ = 0.38), anticipation area (*p* < 0.05, *R*^2^ = 0.43) and empty area (*p* < 0.05, *R*^2^ = 0.22). Fixation durations was higher for successful than unsuccessful strokes: opponent's upper body: 270.0 ± 98.8 ms vs. 176.4 ± 120.4 ms; anticipation area: 402.2 ± 86.7 ms vs. 337.9 ± 156.5 ms and empty area: 364.9 ± 257.0 ms vs. 195.3 ± 126.0 ms.

## Discussion

This is one of the first studies to investigate the effects of fatigue on gaze behavior in a real badminton game setting. Fatigue indicators showed that the experimental procedure successfully induced a similar level of physical fatigue as occurs in real-game conditions. The results only partially confirmed our first hypothesis since acute physical fatigue did not shorten fixation durations, although it did induce a higher number of fixations. However, fatigue did not affect performance. Our second hypothesis was also partially confirmed: fixation durations were longer, not shorter as hypothesized, however the number of fixations was higher for successful strokes compared to unsuccessful strokes.

The lack of an effect of fatigue on performance could be attributed to the novice status of the participants. They won few points and failed a relatively high proportion of strokes both with and without fatigue. The lack of an impact of fatigue on performance could result from the use of other types of anticipatory mechanisms (information pick-up strategies, motor responses or contextual information) to compensate for the fatigued mechanism and maintain results (performance).

This study adds valuable information to the small body of literature in the domain of gaze behavior: the results showed that acute physical fatigue increased the number of fixations on two specific locations (shuttlecock and empty area after opponent's stroke), but did not modify fixation duration. The novice participants demonstrated a random visual strategy to pick-up information because of their low experience level. The increase in the number of fixations on the shuttlecock and empty area after the opponent's stroke suggested that fatigue caused disorganized search behavior. This is consistent with the results of another study in badminton (but not in a real-game setting) that suggested that fatigue negatively impacted operational processes, resulting in a reduction in the efficiency of gaze behavior (Mann et al., 2007). Similarly, during a free-viewing visual search task (non-sports context), incremental exhaustive aerobic exercise increased saccade duration and decreased average saccade velocity (Piras et al., 2017). Together, these results suggest that fatigue has a negative effect on gaze behavior, however further studies in real-game settings, with fatigue conditions like those induced in a real match are required to fully confirm this.

The relationships found between gaze behavior and points won is likely specific to novice players. In contrast with expert players who tend to use tactical information in anticipation of the opponent's stroke (Pluijms et al., 2013), the novice players mainly fixed the boundary lines, with shorter fixations on empty area before their stroke, to win points. Higher numbers of fixations on the boundary lines may be necessary for novice players to integrate the dimensions of the court. This information could be useful, (1) to not return shots made by the opponent that are out of court and (2) to stay on the move during the opponent's stroke so as not to lose time and make a successful return stroke. The shorter fixations on the empty area before their stroke suggests that the novices needed to keep their focus on relevant areas prior to making their stroke. It seems reasonable to hypothesize that they needed a long information intake from the shuttlecock for this purpose.

The novice players in this study fixed all nine AOIs analyzed. Successful strokes were particularly related to four AOIs (opponent's racket, shuttlecock, opponent's upper body, and anticipation area) and were not related to one AOI (boundary lines). This is in line with the finding by Roca et al. (2013) that rather than fixing on a few strategic locations, novice players fix on many locations. However, in accordance with Piras et al. (2017). who found in tennis that novice players focused on racket and ball parts, in the present study players fixed the shuttlecock significantly more than other areas, and tended to fix anticipation area (between opponent's racket and shuttlecock) more often than other locations.

The difference in the areas fixed between the present study and previous studies can be explained by the use of a real-game setting in the present study, rather than video recordings (Sáenz-Moncaleano et al., 2018; Russo and Ottoboni, 2019). In the present study, gaze durations were longer for successful strokes, which we considered as an indicator of accuracy, and fixation duration is positively correlated with accuracy (Shargal et al., 2015; Schapschröer et al., 2016). The longer fixation durations on the opponent's upper body and anticipation area for successful strokes suggested these locations provided relevant information for novices. It is possible that the longer fixation durations recorded for empty area were actually due to simultaneous head and eye movement which reduced the accuracy of the eye tracker. We had hypothesized that fixations on anticipation area would allow information to be collected through parafoveal processing, since the focus was close to the future point of impact between the shuttlecock and racket, near to the opponent's body (Smith et al., 2016). However an extended visual span may only develop with expertise (Triolet et al., 2013), therefore the novice players only used foveal vision on the areas on which they fixed, optimizing the information gathered for their level of ability. Although the participants were novice badminton players, they had experience in other sports. It is possible that they had thus developed and used an extended visual, even if shuttlecock was the most important AOI.

The type of visual search strategy used is a predictor of expertise (Triolet et al., 2013). Studies have shown that expert players fixed on fewer locations for longer durations than novice players (Westfall et al., 2014; Shargal et al., 2015; Van Maarseveen et al., 2018; Brams et al., 2019). In the present study, successful strokes were associated with longer fixation durations and fixations on more locations, which is somewhat similar to gaze behavior used by expert players.

The main limitation of this study was that the eye-tracking device only recorded foveal vision, and did not provide information about peripheral vision or the participant's focus of attention (Abernethy et al., 1993). However, gaze direction and spatial allocation are highly correlated (Williams and Ericsson, 2005), therefore our results may provide an indication of participants' focus of attention. Moreover, saccades are an important gaze behavior that could have been measured in our study. However, the signal detection of saccades was not possible in real-game conditions because of the moving targets and the moving participant wearing the eye-tracking glasses.

This study has several strengths. We attempted to use a robust methodology in terms of eye-tracking research in sports by basing the method on four criteria (Williams et al., 2002): realistic viewing conditions, naturalistic responses, precise measurements and the analysis of large amounts of gaze-data. The recording of data in a real-game setting allowed the perception-action system to be evaluated as a whole, rather than in part as is often the case in controlled studies. Moreover, the analysis of the relationship between fatigue, gaze behavior and performance provided information relating to tactical skills, which are directly related to the perception-action system (Zwierko et al., 2019).

## Conclusion

The results of this study showed that acute physical fatigue impacted the gaze behavior of novice players during a real badminton game setting. The number of fixations on the shuttlecock and on empty area after the opponent's stroke increased with fatigue. Furthermore, two distinct gaze behaviors were found for successful and unsuccessful performance: points won were associated with fixations on the boundary lines and few fixation durations on empty area before the participant's stroke, and successful strokes were related to long fixation durations, few fixation durations on empty area and a large number of fixations on the shuttlecock, racket, opponent's upper body and anticipation area. These results confirm some previous results found in laboratory conditions and provide new data in real-game conditions. The substantial differences in some aspects of gaze behavior between the results of the present study and those of studies that used controlled situations confirm the importance of evaluating gaze behaviors in near real-world conditions.

## Data Availability Statement

The raw data supporting the conclusions of this article will be made available by the authors, without undue reservation.

## Ethics Statement

The studies involving human participants were reviewed and approved by National Ethical Committee, University Paris-Saclay, CER-Paris-Saclay-2019-058. The patients/participants provided their written informed consent to participate in this study.

## Author Contributions

AR, DB, JS, and MLT contributed to conception and design of the study. MLT and LD organized the database. MLT performed the statistical analysis and wrote the first draft of the manuscript. All authors contributed to manuscript revision, read, and approved the submitted version.

## Conflict of Interest

The authors declare that the research was conducted in the absence of any commercial or financial relationships that could be construed as a potential conflict of interest.

## Publisher's Note

All claims expressed in this article are solely those of the authors and do not necessarily represent those of their affiliated organizations, or those of the publisher, the editors and the reviewers. Any product that may be evaluated in this article, or claim that may be made by its manufacturer, is not guaranteed or endorsed by the publisher.
